# Design and test of smashing and scattering device of double-channel feeding ratoon rice harvester

**DOI:** 10.1038/s41598-022-18938-7

**Published:** 2022-09-24

**Authors:** Jianwei Fu, Chao Ji, Weikang Wang, Haopeng Liu, Guozhong Zhang, Yuan Gao, Yong Zhou, Mohamed Anwer Abdeen

**Affiliations:** 1 College of Engineering, Huazhong Agricultural University, Wuhan, 430070 China; 2 Key Laboratory of Agricultural Equipment in Mid-Lower Yangtze River, Ministry of Agriculture and Rural Affairs, Wuhan, 430070 China

**Keywords:** Mechanical engineering, Software, Design, synthesis and processing

## Abstract

Since it is requested that the smashed straw should not be scattered onto the stubble in the first season harvest, a smashing and scattering device matched with the first season harvester of ratoon rice was designed in this paper to smash and scatter the straw into the rolling area. The main structural parameters affecting the straw scattering performance were determined by theoretical analysis of the deflecting performance of the straw deflector. EDEM software was used to simulate and analyze the straw scattering situation under the action of the deflector. Taking the qualified rate of straw scattering as the performance index, the optimal parameter combination of the straw deflector was determined by L_27_ (3^13^) orthogonal test, and the operating performance of the device was verified by bench test and field comparison test. The test results show that: The optimal parameter combination was 45° between the straw deflector and the vertical direction, 200 mm height difference between straw deflector and outlet, 0° inclination angle of inner deflector and 35° inclination angle of outer deflector. The qualified rate of straw scattering was 72.2% in the corresponding simulation test, 93.6% in the bench test and 95.2% in the field test, which could meet the demand of first season harvest of ratoon rice.

Ratoon rice refers to the production of a second rice crop in one cropping season developed from regenerating rice tillers from nodal buds of the stubble that was left behind after the first seasonal rice harvest^1–3^. It can make full use of temperature and light resources and save labor and seed. In addition, with high quality, the ratoon rice can help to increase both production and income^4–6^.

In order to increase the annual yield of ratoon rice, high yield in the first season of ratoon rice and full heading in the regenerating season are required^7,8^. The yield of the first season is mainly determined by rice varieties, growth conditions (light, temperature, water, fertilizer, etc.) and cultivation and management measures, while the yield of the second season is determined by the harvest, growth conditions and management measures of the first season^9,10^. Agricultural studies show that during mechanized harvesting in the first season of ratooning rice, the paddy is wet and soft, and the rice stems are thick and green. In order to promote the sprouting of ear heads in the ratooning season, it is required to have high stubble retention, low rolling rate and light rolling degree, and strong threshing capacity of the roller to adapt to strong and wet materials. When the harvester is working, miscellaneous residues such as rice straw and leaves will be scattered into the paddy field. If the straw is smashed and scattered in disorder, they will remain on the upper part of the rice stubble, which will easily cause rice straw to rot and thus affect the germination of regenerated ear heads^11,12^. Therefore, the first season harvest of ratoon rice should make sure that the straw is smashed and scattered without covering the stubble.

The harvest technology in the first season of ratoon rice is still bottleneck in the popularization and development of ratoon rice^13,14^. There are few studies on harvesting technology of ratoon rice in the first season, and no corresponding straw processing techniques have been found. Scholars at home and abroad have carried out researches on increasing the rotational speed of the smashing tool shaft, improving the structure of the smashing tool, improving the air flow characteristics in the smashing chamber, improving the shape of the smashing chamber, installing the scattering regulating device and so on to increase the evenness of the straw scattering of the smashing and scattering returning machine.

Zhang et al.^15^ designed an adjustable straw smashing and scattering returning machine, whose qualified rate of smashed straw length could reach 90.01% and the unevenness of straw scattering was 22.95%. Sun et al.^16^ designed a differential saw-cutting rice straw smashing and returning machine. Its qualified rate of straw length could reach 93.23% and the unevenness of scattering was 20.89%. Qin et al.^17^ designed a straw smashing and scattering device for the straw returning fertilizing and sowing machine. Its qualified rate of straw length was 96.6%, the qualified rate of straw scattering range was 90.2%, and the unevenness of straw scattering was only 11.9%. The qualified rate of straw smashing length and the unevenness of straw scattering are both used as the performance indexes of the existing straw smashing and scattering devices matching with harvesters. Thakur et al.^18^ developed a smashing device matching with harvester, which achieved the best performance when the stem moisture content was 70%, machine tool working speed was 0.56 m/s and the knife roller speed was 1500 r/min. Schillinger et al.^19^ installed a high-pressure fan on the harvester to improve the scatter evenness of straw. However, although the fan could improve the evenness of the straw scattering after smashing, it would make the structure of the machine complex, and significantly increase the power consumption of the machine. The straw scatter width and evenness also become more difficult to control. Lisowski et al.^20^ used Fluent software to analyze the movement process of the straw at the outlet of the harvester cylinder, and studied the relationship between the speed of the smashing knife of the smashing device and the outlet speed of the threshing cylinder. These researches mainly took straw counters-field set or regular harvester as research objects, whose performance indexes such as average stubble height, qualified rate of crushing length, scattering unevenness, and scattering width were given priority to. Although these researches improved straw crushing effect and evenness of straw scattering, they did not meet the requirements of mechanized agricultural harvest of ratoon rice in the first season and could not effectively solve the problem of straw covering during the first harvest of ratoon rice, which directly affected the growth of regenerated buds in the second season.

To effectively solve the straw covering problem during harvest of ratoon rice in the first season, based on the double-channel feeding ratoon rice harvester independently developed by our team, we adopted the EDEM software to simulate the straw scattering process to obtain the optimal structural parameters of the straw deflector which can lead the crushed straw into the rolling area. At the same time, bench and field experiments were carried out to verify the feasibility of the design. This study can provide reference for the research and promotion of mechanized harvesting technology and equipment of ratoon rice in the middle and lower reaches of the Yangtze River.

## Materials and methods

### The structure of smashing and scattering device

**Figure 1:**
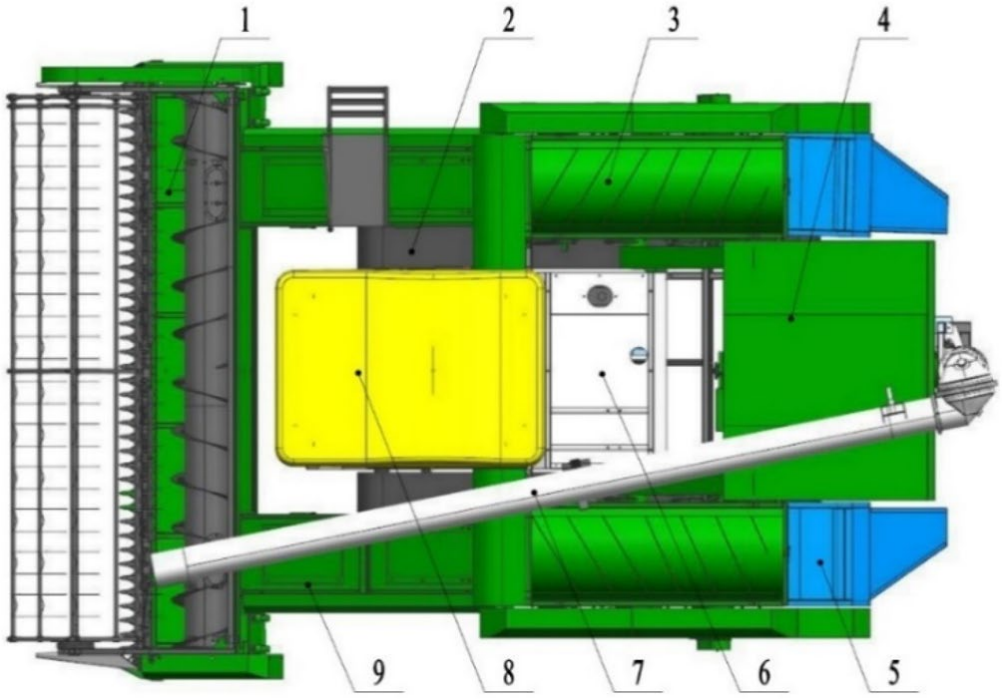
Double-channel feeding ratoon rice harvester. (**1**) double-channel header, (**2**) track, (**3**) threshing and cleaning device, (**4**) grain bin, (**5**) smashing and scattering device, (**6**) engine, (**7**) grain unloading device, (**8**) sunshade, (**9**) conveying channel. (SolidWorks 2020, https://www.solidworks.com/zh-hans).

The smashing and scattering device was fit on the feeding ratoon rice harvester^21,22^, which is shown in Fig. 1. The double-channel feeding ratoon rice harvester is a harvester independently developed by our research team for the harvest of ratoon rice in its first season, whose feeding amount is 4.0 kg/s, cutting width is 3000 mm, and supporting power is 65 kW. The whole machine consists of a crawler chassis, a header, two sets of threshing and cleaning devices symmetrically arranged on the left and right, a straw scattering device, a grain bin, and a power and transmission system. When the harvester is working, the rice plants above the stubble height are cut off by the cutter and fall into the header, which are pushed to the left and right feeding inlets respectively by the auger, and fed to the following two sets of threshing and cleaning devices by the chain scraper conveyor. The clean grain after threshing, separation and impurity removal in the threshing and cleaning room is centrally led into the grain bin by the chain scraper conveyor. The straw is crushed by the smashing and scattering device at rear and scattered to the two crawler rolling areas. Finally, the grain in the grain bin is unloaded by the grain unloading tube to complete the harvest operation.

**Figure 2:**
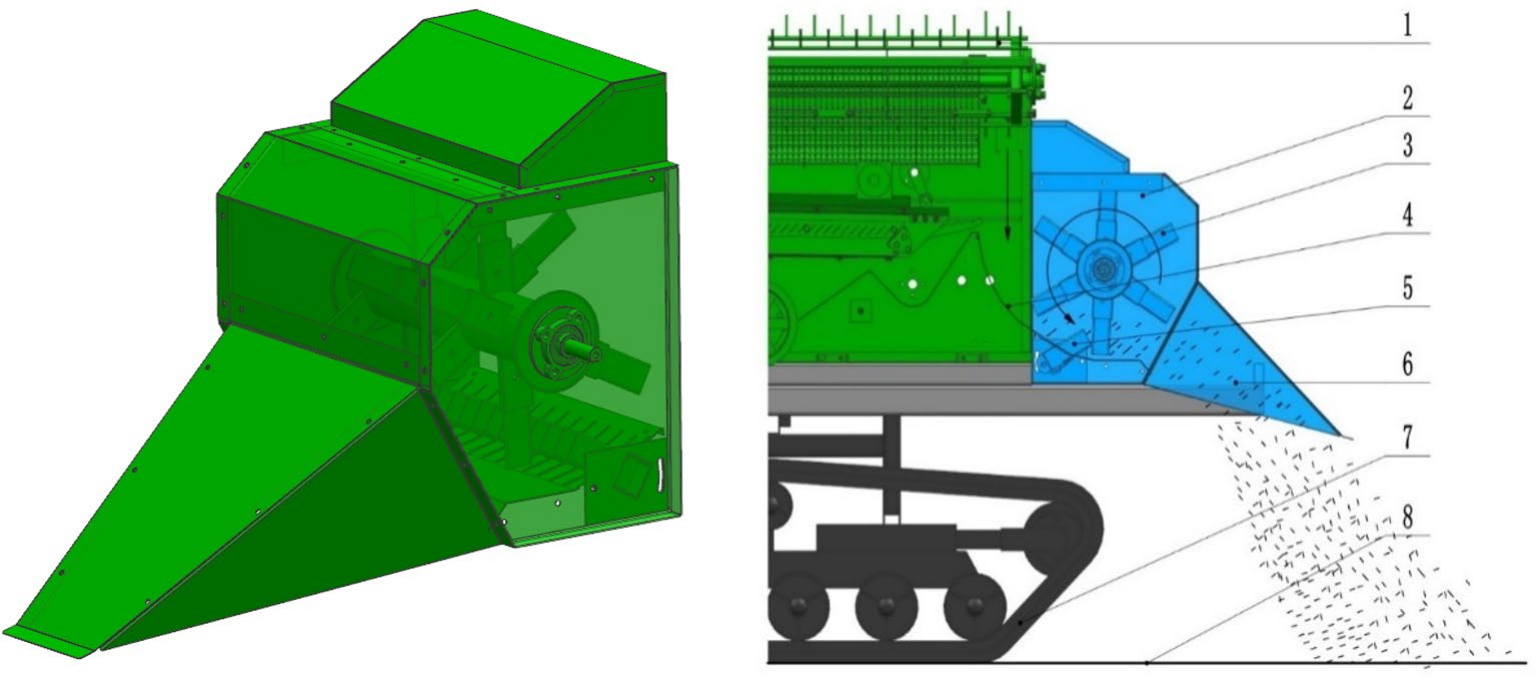
Structure and schematic diagram of smashing and scattering device. (**1**) threshing cylinder, (**2**) main box of the smashing and scattering device, (**3**) moving knife roller, (**4**) arc straw catching plate, (**5**) fix knife set, (**6**) straw deflector, (**7**) chassis, (**8**) ground. (SolidWorks 2020, https://www.solidworks.com/zh-hans).

The smashing and scattering device mainly consist of main box, straw deflector, knife roller, fix knife set and so on. The main box is connected by hinges to the outlet of the threshing and cleaning cylinder of the harvester, from which the straw is discharged. The side cover plate of the main box is provided with an anti-entangling ring, which is compatible with the anti-entangling end cover on the knife shaft of the moving knife roller to avoid the straw winding the knife shaft. There is an arc straw catching plate between the smashing and scattering device and the threshing and cleaning chamber. The straw falling on the arc straw catching plate is grabbed by the high-speed rotating knife roller and enters the box of the smashing and scattering device. Under the combined action of multiple groups of moving knives and fixed knives, the straw is smashed into fragmentized ones. Under the action of high-speed rotating centrifugal force of the knife roller, the smashed straw is thrown out along the tangent direction. Finally, under the action of flow diversion of the straw deflector, the smashed straw is thrown to the two crawler rolling areas respectively. The structure and working principle of smashed straw are shown in Fig. 2.

### Deflect performance of straw deflector

The working principle of the straw deflector is to change the motion state of the straw after colliding with the deflector under the external force which will change its initial position, angle, and velocity, allowing it continue to move by a new trajectory. The straw deflector consists of cover deflector, inner deflector, and outer deflector. The force states when the smashed straw collides with different deflector are similar. Taking the straw cover deflector as an example, the force at the moment when straw collides with the cover deflector was analyzed. The object was a piece of single smashed straw, which was regarded as a particle, and the influence of air flow on the straw was ignored.

**Figure 3:**
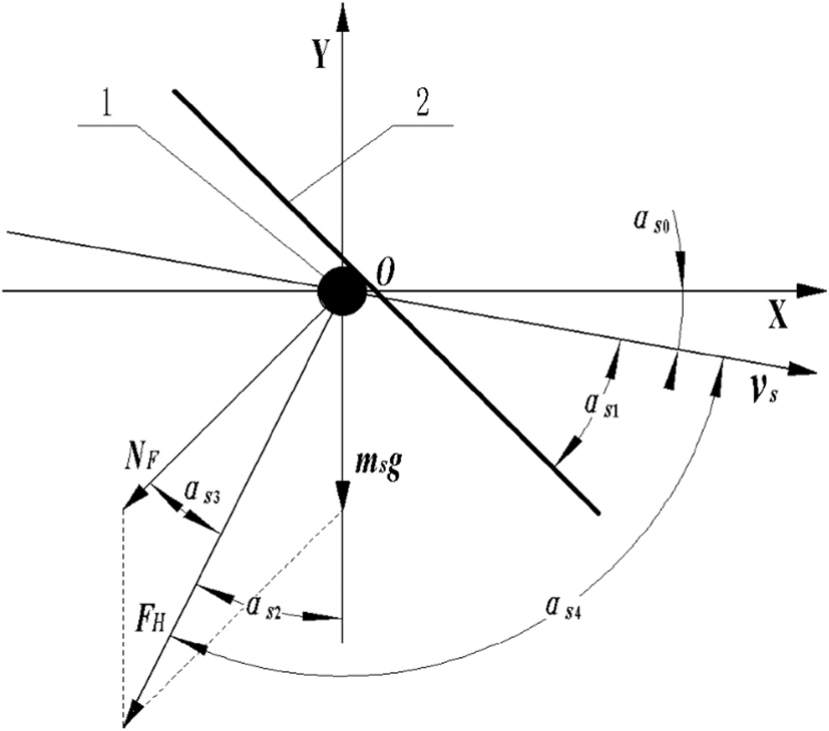
Analysis of deflect principle for straw deflector. (**1**) smashed straw, (**2**) straw deflector. *Note*: msg is the mass of the smashed straw, N; NF is the reactive force to smashed straw when it collides with the deflector, which is perpendicular to the deflector, N; FH is the resultant force of msg and NF, N.

Take the smashed straw as the origin of the coordinate axis, the motion direction of the straw as the positive X-axis direction, and the opposite gravity direction as the positive Y-axis direction to establish the plane coordinate system as shown in Fig. 3. By analyzing the instantaneous force state of the straw deflector when the smashed straw collided with it, it can be obtained:

1 $$ \alpha_{S4} + \alpha_{S0} = \alpha_{S2} + 90^\circ $$

It can be obtained from the triangle similarity relation:

2 $$ \left\{ {\begin{array}{*{20}l} {\alpha_{S0} + \alpha_{S1} = \alpha_{S2} + \alpha_{S3} } \hfill \\ {\tan \alpha_{S2} = \frac{{N_{F} \cos \left( {\alpha_{S2} + \alpha_{S3} } \right)}}{{m_{S} g + N_{F} \sin \left( {\alpha_{S2} + \alpha_{S3} } \right)}}} \hfill \\ \end{array} } \right. $$

Where *v*_*s*_ = Instantaneous speed of the straw colliding with the deflector (m/s), *m*_*s*_ = Mass of the smashed straw (kg), *g* = Gravitational acceleration (9.8 m/s^2^), *α*_*s*0_ = The angle between the direction of motion and the X axis when colliding with the deflector (°), *α*_*s*1_ = The angle between the direction of motion and the cover deflector when colliding with the deflector (°), *α*_*s*2_ = The angle between *F*_*H*_ and negative Y-axis direction when the straw colliding with the deflector (°), *α*_*s*3_ = The angle between *F*_*H*_ and *N*_*F*_ when the straw colliding with the deflector (°), *α*_*s*4_ = The angle between the direction of motion and the *F*_*H*_ when the straw colliding with the deflector (°).

It can be obtained by substituting Eq. (1) into Eq. (2):

3 $$ \alpha_{S4} = 90^\circ - \alpha_{S0} + \arctan \frac{{N_{F} \cos (\alpha_{S0} + \alpha_{S1} )}}{{m_{S} g + N_{F} \sin (\alpha_{S0} + \alpha_{S1} )}} $$

It can be seen from Eq. (3) that when the speed direction of the straw colliding with the straw deflector is constant, the change of the angle of the straw deflector will make a difference to the motion trajectory of the straw at the next moment; at the same time, due to the different circumfluence position and initial velocity when the straw leaves the knife roller, the collision position of the straw will be different in the Y-axis direction when it collides with the deflector. Therefore, the angle and size of the straw deflector have a great influence on the scattering performance of the straw.

### Parameter design of straw deflector

**Figure 4:**
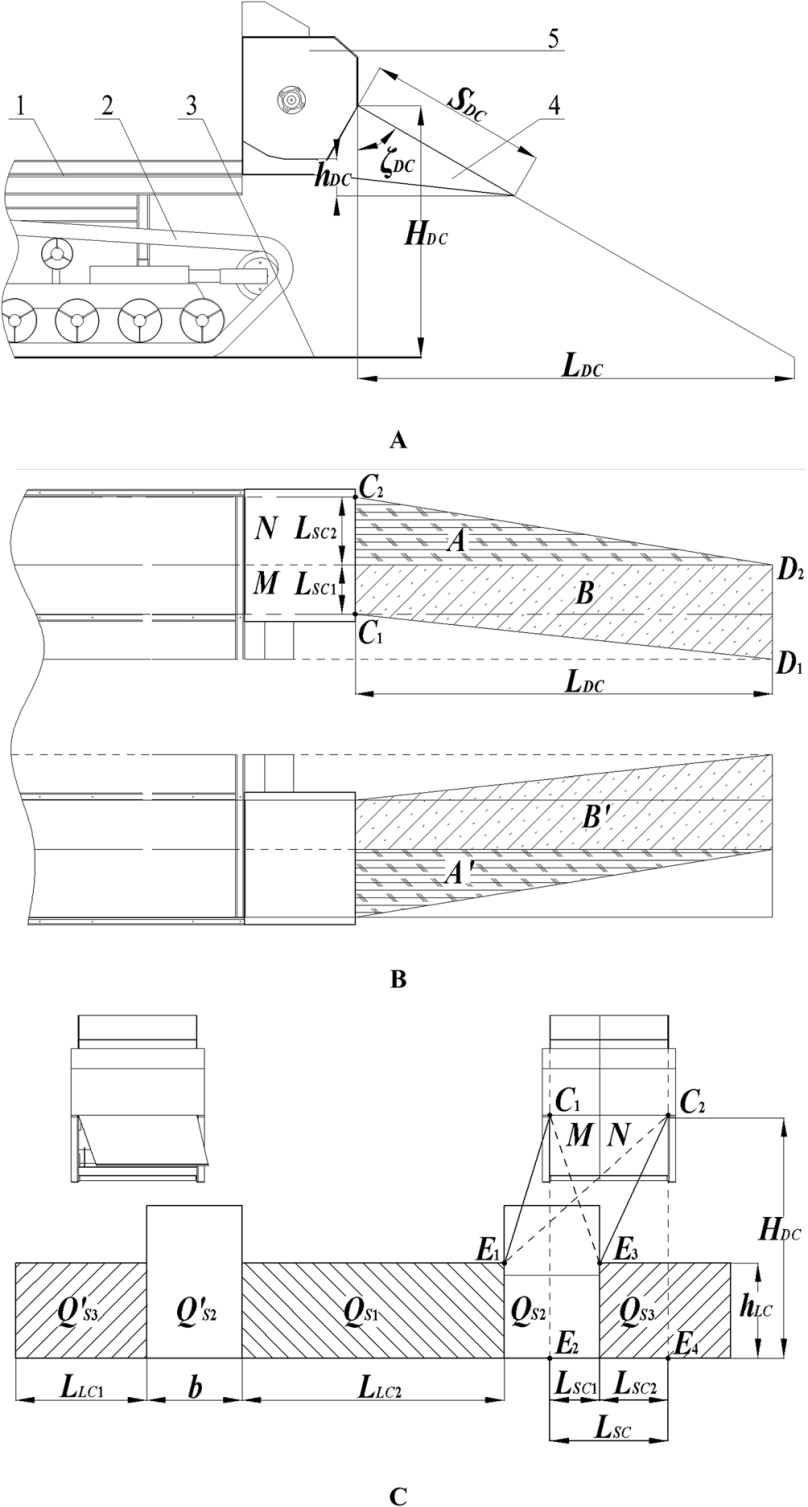
Diagram of smashed straw throwing. (**A**) Lateral view, (**B**) Top view, (**C**) Front view; (**1**) frame, (**2**) track, (**3**) ground, (**4**) straw deflector, (**5**) main body of the scatter box. *Note*: *L*_*SC*_ is the width of the smashing and scattering device, mm; *L*_*SC*1_ is the width of the scatter device above the track, mm; *L*_*SC*2_ is the width of the scatter at the lateral of the track, mm; *L*_*DC*_ is the scattering distance of the smashed straw, mm; *L*_*LC*1_, *L*_*LC*2_ is the width of the stubble area at the outer side of the track, mm; *S*_*DC*_ is the length of the cover deflector, mm; *ζ*_*DC*_ is the angle between the cover deflector and the vertical direction, °; *h*_*DC*_ is the height difference between the tail of the cover deflector and the outlet, mm; *h*_*LC*_ is the stubble height, mm; *H*_*DC*_ is the height from the top spot of the outlet to the ground, mm.

Figure 4 is the diagram of smashed straw scattering. From the structural parameters of the whole machine, the positional relationship between the smashing and scattering device and the track of the double-channel feeding ratoon rice harvester is shown in Fig. 4B, C. The width of the smashing and scattering device is *L*_*SC*_. Only part of the device is located directly above the track with a width of *L*_*SC*1_, corresponding to the space M inside the device; the rest part is located outside the track with a width of *L*_*SC2*_, corresponding to the space N inside the device.

Ignoring the air force and interaction between the straw, the straw is only subjected to air resistance and gravity. According to the agronomy requirements of the first season harvest of ratoon rice, the straw discharged from the harvester should not cover the rice stubble, that is, the ideal state is to scatter all the smashed straw to the rolling area. As shown in Fig. 4B, A is the scattering area generated from N space under the action of the outer deflector, and B is the scattering area generated in M space under the action of the inner deflector. It can be seen the smaller the A area is, the closer it is to the ideal state. If there is no straw deflector, the smashed straw will fall onto the ground after leaving the knife, all the straw from M space will be discharged to zone B, and all the straw from N space will be discharged to zone A.

After installing the straw deflector, the scatter distance of smashed straw can be limited. As shown in Fig. 4A, *ζ*_*DC*_ is the angle between the straw deflector and the vertical direction. The larger *ζ*_*DC*_ was, the farther the straw will be scattered. When *ζ*_*DC*_ ≥ 90°, the straw deflector loses its function of deflecting. Besides, the larger *ζ*_*DC*_ is, the larger the length of cover deflector *S*_*DC*_ will be. When ζ = 0°, the smashed straw will collide with the vertical baffle and fall directly, so it could not be scattered backward and will easily get blocked in the chamber. In the Actual scattering process, due to the influence of air, the farther the scattering distance is, the farther the lateral movement of smashed straw will be, that is, the amount of smashed straw scattered outside the rolling area will increase. In order to explore the influence of *ζ*_*DC*_ on the qualified rate of the scattering, the *ζ*_*DC*_ test factors are set as 30°, 45° and 60° considering the length of the straw cover deflector *S*_*DC*_ and the actual blockage.

In Fig. 4A, *h*_*DC*_ is the height difference between the tail of the straw deflector and the outlet. The smaller *h*_*DC*_ is, the fewer smashed straw will collide with the straw deflector after flat, up, or down scattering. In order to explore the impact of *h*_*DC*_ on the qualified rate of scattering, the factor levels were set as 0 mm, 100 mm, and 200 mm on the premise of not interfering with other mechanisms fully considering the structural relations between straw deflector and frame, grain bin, and chassis track.

As shown in Fig. 4C, the stubble between and beside the two is not rolled when harvesting, i.e., *Q’ *_*S3*_, *Q*_*S1*_ and *Q*_*S3*_, and the stubble height is *h*_*LC*_. It can be seen the maximum height of the boundary between *Q*_*S1*_ and *Q*_*S3*_ should be avoided for the straw discharged from M and N zones to falling into the rolling area *Q*_*S2*_. Therefore, it can be inferred according to the geometric relationship that the following conditions must be met for the inner and outer deflector to scatter the smashed straw into the rolling area rather than on the rice stubble:

4 $$ \left\{ {_{{\angle E_{3} C_{2} E_{4} \le \beta_{y} \le \angle E_{1} C_{2} E_{4} }}^{{ - \angle E_{2} C_{1} E_{3} \le \beta_{z} \le \angle E_{2} C_{1} E_{1} }} } \right. $$

Where *β*_*z*_ = inclination angle of inner deflector (°), *β*_*y*_ = inclination angle of outer deflector (°).

It can be obtained from the whole machine and the structural parameters of the smashing chamber that *β*_*z*_ ∈ [− 14°,13°], *β*_*y*_ ∈ [23°,48°]. Therefore, the level of inclination factor of the inner deflector was set as − 10°, 0° and 10°, and the level of inclination factor of the outer deflector was set as 25°, 35° and 45°.

### Simulation test of straw scattering process based on EDEM

#### Simulation model and parameter design

Pro/E software was used to carry out three-dimensional modeling of the smashing and scattering device and imported it into the geometry module in EDEM software^23,24^. The simulated gravity acceleration was 9.81 m/s^2^, and the material of the smashing and scattering device was selected as Q235. The front of the smashing chamber may give the straw a wrong way out, so it was closed in the 3D model during simulation.

**Figure 5:**
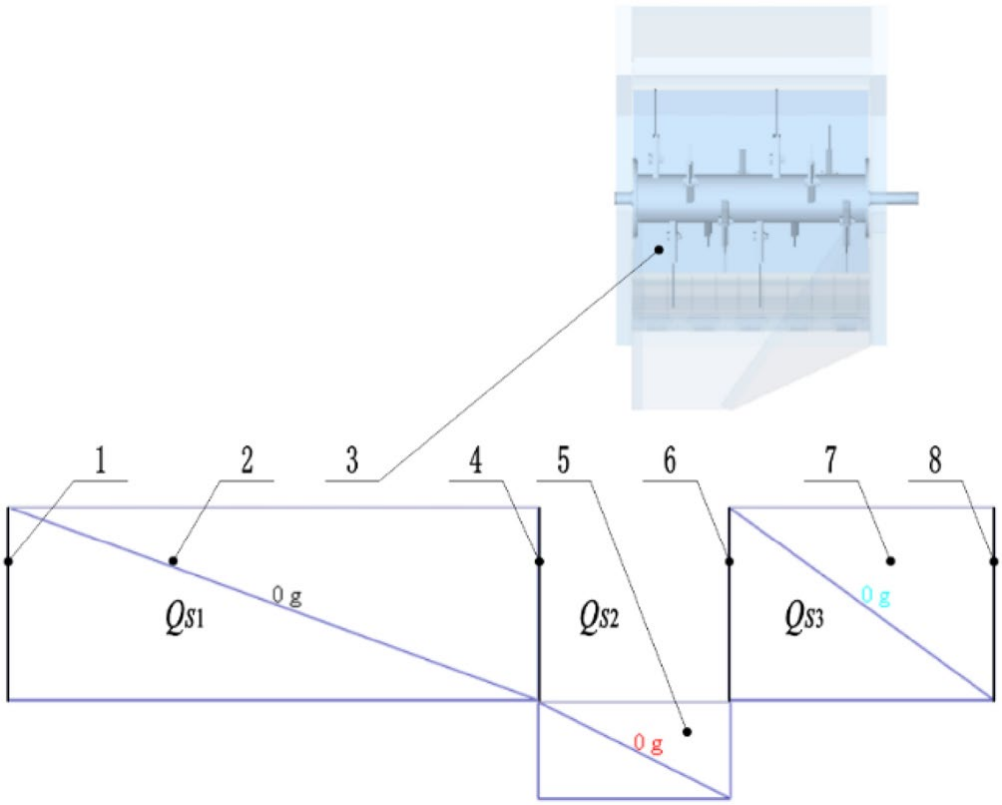
Simulation sensors setup. (**1**) baffle I, (**2**) square sensor I, (**3**) smashing and scattering device, (**4**) baffle II, (**5**) square sensor II, (**6**) baffle III, (**7**) Square sensor III, (**8**) baffle IV.

As shown in Fig. 5, the stubble between and beside the two tracks of the double-channel feeding ratoon rice harvester needs to be kept for the ratoon rice, thus the smashed and scattered straw should be discharged into the rolling area rather than these areas. Therefore, four baffles with a height of 350 mm whose material is straw were arranged in the model. The two baffles in the middle were 400 mm apart to simulate the rolling area, and the other two baffles simulate the stubble boundary, thus three regions were separated out, which is *Q*_*S*1_, *Q*_*S*2_, and *Q*_*S*3_ in Fig. 5. In the simulation, the Hertz-Mindlin contact model without sliding was set among particles and between particles and geometric model^25^.

A square mass sensor II with a width of 400 mm was set on the rolling area, which was used to calculate the accumulative mass m_2_ of straw falling into the area in continuous 3 s when the straw was scattered through the device. Then, square mass sensor I and square mass sensor III were set respectively, and the mass of straw scattered on the rice stubble in the two areas of the harvest area was marked as m_1_ and m_3_.

The feeding amount of the double-channel feed ratoon rice harvester is 4.0 kg/s, 2.0 kg/s for a single channel. The straw fed into the smashing device was set to be 1 kg/s according to the ratio of grain and straw which is 1:1. During each simulation, the knife roller was set to rotate continuously with the speed of 2800 r/min, the total mass was set to be 3 kg, and 1 kg was generated per second. The total simulation time was set to 5 s to reduce the straw residue in the chamber. The particle generating plane was arranged above the arc straw catching plate, and the length and width of the rectangular plane were 496 mm and 150 mm respectively. According to the linear velocity of threshing cylinder, the particles were set to be − 21 m/s along Z axis.

**Figure 6:**
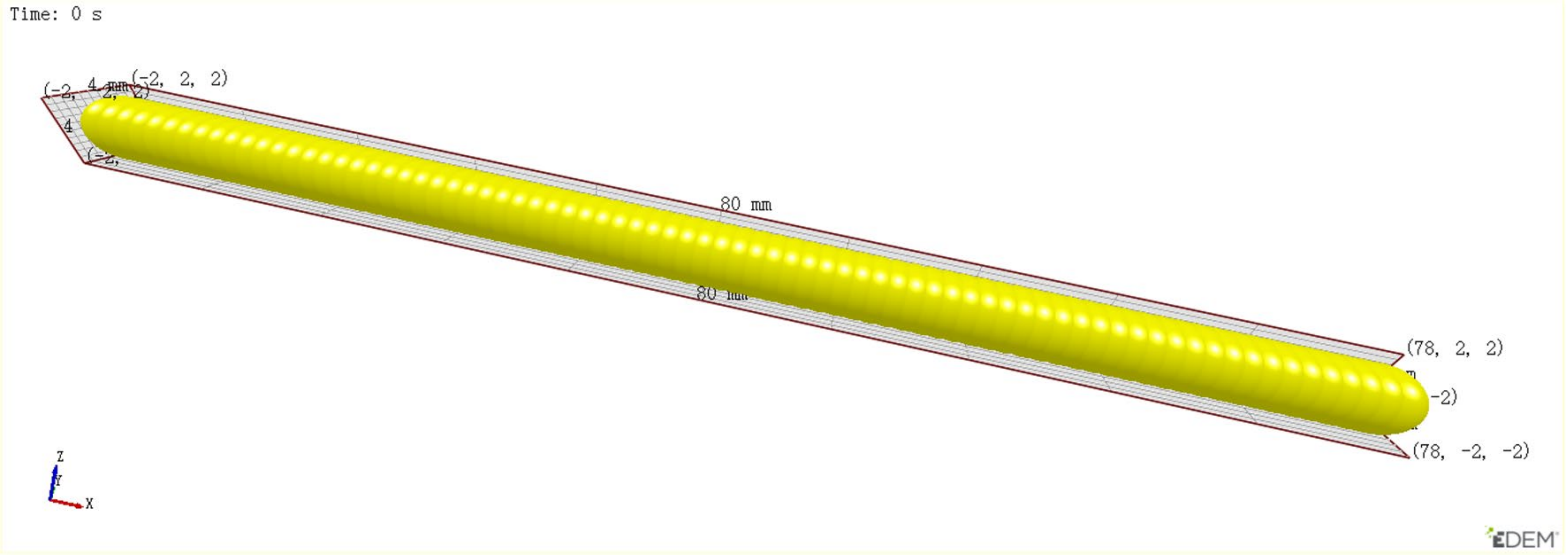
Straw discrete element model.

The straw material was approximately formed into a cylinder with a diameter of 4 mm and a length of about 80 mm to build the straw discrete element model of ratoon rice, as shown in Fig. 6. Simulation parameters of the straw are shown in Table 1^26,27^.

**Table 1:** Simulation parameters.

Parameters	Value
Poisson’s ratio of the straw	0.4
Density of the straw/(kg/m^3^)	229
Shear modulus of the straw/(MPa)	9.1
Poisson’s ratio of Q235	0.3
Density of Q235/(kg/m3)	7850
Shear modulus of Q235/(MPa)	80,000
Straw-straw coefficient of restitution	0.3
Straw-straw static friction coefficient	0.3
Straw-straw rolling friction coefficient	0.01
Straw-Q235 coefficient of restitution	0.3
Straw-Q235 static friction coefficient	0.3
Straw-Q235 rolling friction coefficient	0.01

#### Simulation test design

Since there will be straw residue in the smashing chamber during simulation, and a small amount of straw will fall outside the statistical area, the sum of the mass statistics in the three square sensors is taken as the total amount of straw. By counting the mass of the straw falling into the three square sensors m_1_, m_2_ and m_3_, the proportion of the straw falling into the smashing area *η*_*FZ*_ was calculated as:

5 $$ \eta_{FZ} = \frac{{m_{2} }}{{m_{1} + m_{2} + m_{3} }} \times 100\% $$

Where *η*_*FZ*_ = Qualified rate of scattered smashed straw in the simulation (%), *m*_1_ = Total mass of smashed straw in square sensor I (simulated stubble area Q_S1_) (g), *m*_2_ = Total mass of smashed straw in square sensor II (simulated stubble area Q_S2_) (g), *m*_3_ = Total mass of smashed straw in square sensor III (simulated stubble area Q_S3_) (g).

*η*_*FZ*_ is used as a parameter to evaluate the performance of straw smashing and scattering device. The higher the value of *η*_*FZ*_ is, the better the performance is. Therefore, it is defined as the qualified rate of straw scattering. The scattering effect of straw under different structural parameters of straw deflector can be analyzed by *η*_*FZ*_.

In this simulation test, the angle between the cover deflector and the vertical direction A, the height difference between the tail of the cover deflector and the outlet B, the inclination angle of the inner deflector C and the inclination angle of the outer deflector D were selected as the test factors. According to the design and analysis of the parameters of the tail of the cover deflector, three levels of the four factors were selected for orthogonal test respectively, as shown in Table 2.

**Table 2:** Test factor level.

Level	Factors			
	A/°	B/mm	C/°	D/°
1	35	0	− 10	25
2	45	100	0	35
3	60	200	10	45

Interaction is an influencing factor to be considered in the process of orthogonal test design. The interactive discrimination tests between factors A and B, A and C, A and D, B and C, B and D, C and D were carried out respectively. Taking the discriminant method of interaction between factors A and B as an example, there were 9 combinations between factor A and B, keeping C and D unchanged. Taking the qualified rate of straw scattering as the index, each combination was tested once, and the same went for the other 5 groups.

According to the generated interaction curve, if the three curves in each combination test have similar trends, it suggested there was no interaction between the two factors, or the interaction could be ignored^28,29^. Finally, the interaction column was set up in the orthogonal table based on the interaction discriminant results and the header of the orthogonal test was designed.

### Bench verification test

The structure model of the straw deflector was constructed by the combination of the optimal structural parameters in the simulation test. The straw deflector was installed on the smashing and scattering device and fit with the double-channel feeding ratoon rice harvester.

The test material was first season ratoon rice at maturity, and the variety was *Fengliangyouxiang* No. 1. The stubble height above 350 mm was manually harvested and transported to the test base. Then it was threshed for the test.

**Figure 7:**
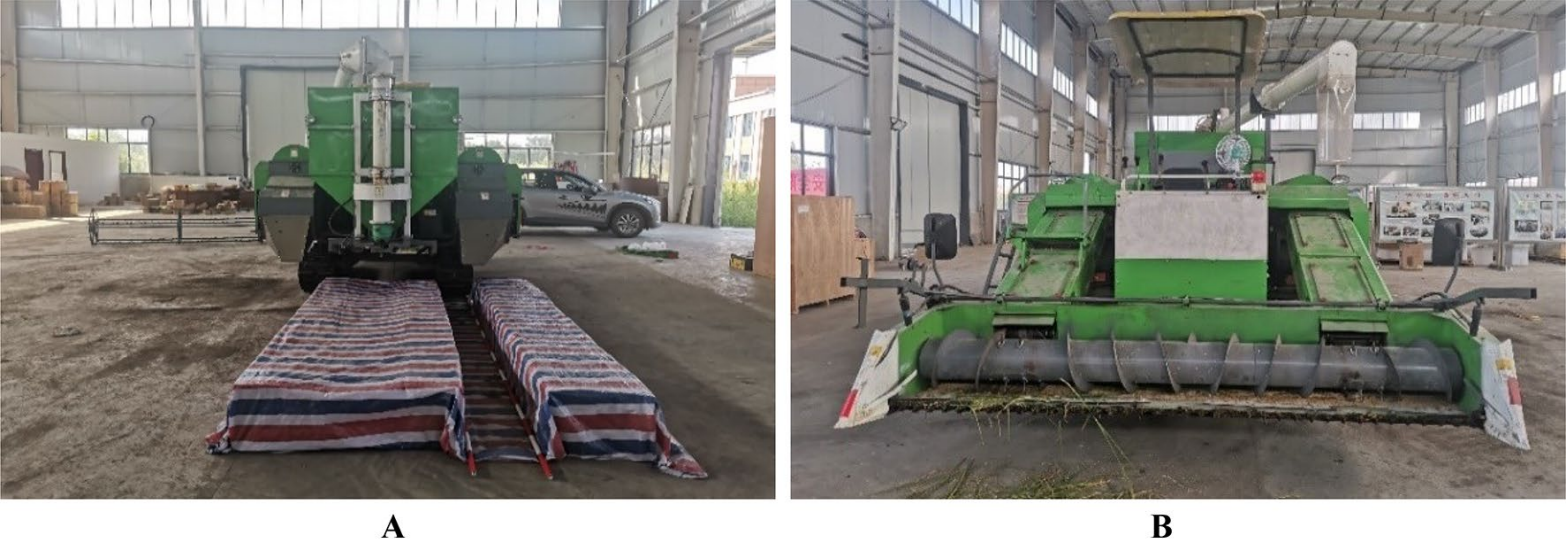
Bench test of smashed straw scattering. (**A**) simulation of rolling and stubble area, (**B**) feeding inlet.

In order to make the test conditions close to the simulation test, the right side (rear view) straw scattering device of the double-channel feeding ratoon rice harvester was selected to install the straw deflector with the optimal parameters obtained from the simulation test. A 400 mm wide and 5000 mm long space is isolated behind the harvester track with color strip cloth to simulate the track rolling area Q_S2_; Stubble area Q_S3_ was between the right boundary of header and the outside of right track; The stubble area Q_S1_ is simulated between the inside of the left track and the inside of the right track, and the stubble height is 350 mm. The material was only fed from the header feeding inlet at the corresponding side of the test side of the straw smashing and scattering device. The reel was removed and the header was lifted to a height of 350 mm above the ground. To ensure safety, during the test, only the working clutch on the test side of the harvester was in the “on” state, the working parts on the other side were not started, and the working clutch was in the “off” state. Thus, only the two-way screw auger of the header rotated, and the cutter was stationary. The test site is shown in Fig. 7.

During the test, 1 kg material was fed from the feeding inlet of the header every second for 3 consecutive times, and the harvester was stopped when the smashing and scattering device stopped scattering the smashed straw at the test side. The straw in three areas behind the tracks was collected. Remove the impurities and weigh the rest M_QS1_, M_QS2_ and M_QS3_. The qualified rate *η*_*QS*_ of the straw was calculated. Repeat the test for 5 times to obtain the average value, then the solution formula of *η*_*QS*_ was:

6 $$ \eta_{QS} = \frac{{M_{QS2} }}{{M_{QS1} + M_{QS2} + M_{QS3} }} \times 100\% $$

Where *η*_*QS*_ = Qualified rate of smashed straw scattering in the bench test (%), M_*QS*1_ = The total mass of smashed straw in simulated stubble area Q_S1_ in the bench test (g), M_*QS*2_ = The total mass of smashed straw in simulated stubble area Q_S2_ in the bench test (g), M_*QS*3_ = The total mass of smashed straw in simulated stubble area Q_S3_ in the bench test (g).

### Field test on scattering performance of smashed straw

**Figure 8:**
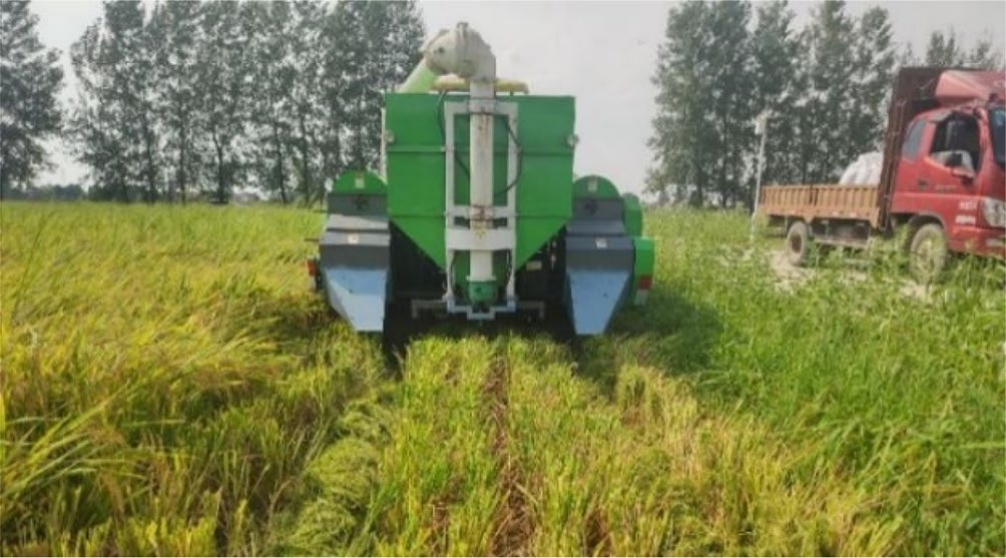
Field test of smashing and scattering device.

To further verify the rationality of simulation test and bench test, the smashing and scattering field performance test of the straw smashing and scattering device, which was installed on the double-channel feeding harvester for ratoon rice, was carried out in the rice demonstration base of Si Wu Men Village, Wulin Town, Honghu City, Hubei Province on August 10th, 2020. The ratoon rice variety is *Fengliangyouxiang 1*, and the field test site is shown in Fig. 8.

Field test was carried out on the scattering performance of the smashing and scattering device based on *Conservation tillage equipment-Smashed straw machine* (GB/T 24675.6-2009)^30^ and the qualified rate of scattering was taken as the test index.

The double-channel feeding ratoon rice harvester operated at a speed of 0.8 m/s testing two strokes. Select 3 points at equal intervals in the two strokes for measurement, and a total of 6 points were tested. Each point was an area with a length of 6 m (i.e., two widths) and a width of 1 m in two adjacent operation strokes. Collect all the straw in the 4 rolling rows in the area, remove the impurity, weight, and record as *M*_*SP1*_; Collect all the straw in the un-rolling stubble area, including all the straw fragments covering the stubble and falling to the ground between the stubble, remove the impurity, weigh, and record as *M*_*SP2*_. The qualified rate *η*_*PS*_ of field integrated scattering can be calculated as:

7 $$ \eta_{PS} = \frac{{M_{SP1} }}{{M_{SP1} + M_{SP2} }} \times 100\% $$

Where *η*_*PS*_ = Qualified rate of integrated scattering in the field (%), *M*_*SP*1_ = The mass of the straw in the rolling area (g), *M*_*SP*2_ = The mass of the straw in the stubble area (g).

### Ethical statement

Human ethics was not required for this study since human were only the operators of the machines rather than the subjects of the test. The collected plant materials and research activities were in accordance with the laws and regulations of Hubei Province, China. The collection of ratoon rice straw materials had been approved by the paddy field owner of Chunlu Specialized Cooperative in Honghu, Hubei, China. All participants gave informed consent prior to participation.

## Results and discussion

### Simulation test results and analysis

#### Interaction discrimination

**Figure 9:**
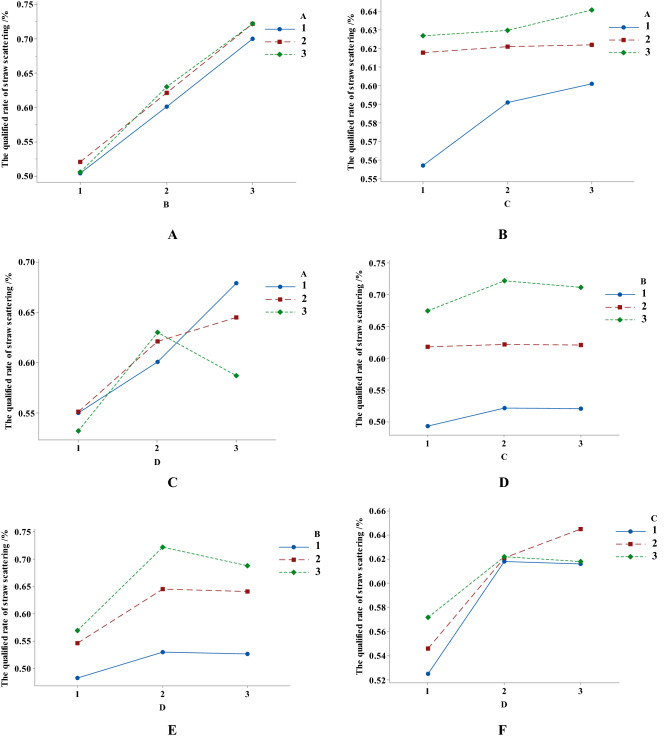
Simulation test results of interactive discriminant between factors. (**A**) **A** and **B**, (**B**) **A** and **C**, (**C**) **A** and **D**, (**D**) **B** and **C**, (**E**) **B** and **D**, (**F**) **C** and **D**.

Figure 9 is the interaction diagram among various factors, in which (a), (b), (d) and (e) curves have basically the same changing trend, indicating that there is no obvious interaction between A and B, A and C, B and C, and B and D. The three curves in (c) and (f) have inconsistent changing trends, indicating that there may be certain interaction between A and D, and between C and D.

### Interaction discrimination

#### Range analysis

There is interaction between factors A and D, C and D in the structural parameters of straw deflector, so the interaction column is set in the orthogonal table. The interaction between any 2 factors with 3 levels occupies two columns, so L_27_ (3^13^) orthogonal table is selected for this test for 8 factors with 3 levels.

Orthogonal test was carried out with L_27_ (3^13^) header, and the simulation test results were obtained for 27 times, as shown in Table 3. According to the evaluation criteria of scattering performance, the higher the qualified rate *η*_*FZ*_ is, the better the scattering effect was. Therefore, the order of primary and secondary factors affecting the scattering performance is B, D, C, A × D, C × D, A.

**Table 3:** Orthogonal test results.

No	Factors													Index
	A	B	(C × D)_2_	vacant	C	vacant	vacant	(A × D)_2_	D	(A × D)_1_	vacant	vacant	(C × D)_1_	*η*_*FZ*_/%
1	1	1	1	1	1	1	1	1	1	1	1	1	1	38.8
2	1	1	1	1	2	2	2	2	2	2	2	2	2	50.4
3	1	1	1	1	3	3	3	3	3	3	3	3	3	51.8
4	1	2	2	2	1	1	1	2	2	2	3	3	3	55.7
5	1	2	2	2	2	2	2	3	3	3	1	1	1	67.9
6	1	2	2	2	3	3	3	1	1	1	2	2	2	53.2
7	1	3	3	3	1	1	1	3	3	3	2	2	2	70.4
8	1	3	3	3	2	2	2	1	1	1	3	3	3	56.9
9	1	3	3	3	3	3	3	2	2	2	1	1	1	68.4
10	2	1	2	3	1	2	3	1	2	3	1	2	3	49.3
11	2	1	2	3	2	3	1	2	3	1	2	3	1	53.0
12	2	1	2	3	3	1	2	3	1	2	3	1	2	47.0
13	2	2	3	1	1	2	3	2	3	1	3	1	2	61.6
14	2	2	3	1	2	3	1	3	1	2	1	2	3	54.6
15	2	2	3	1	3	1	2	1	2	3	2	3	1	62.2
16	2	3	1	2	1	2	3	3	1	2	2	3	1	54.8
17	2	3	1	2	2	3	1	1	2	3	3	1	2	72.2
18	2	3	1	2	3	1	2	2	3	1	1	2	3	62.1
19	3	1	3	2	1	3	2	1	3	2	1	3	2	49.6
20	3	1	3	2	2	1	3	2	1	3	2	1	3	47.1
21	3	1	3	2	3	2	1	3	2	1	3	2	1	50.9
22	3	2	1	3	1	3	2	2	1	3	3	2	1	50.8
23	3	2	1	3	2	1	3	3	2	1	1	3	2	63.0
24	3	2	1	3	3	2	1	1	3	2	2	1	3	56.4
25	3	3	2	1	1	3	2	3	2	1	2	1	3	71.6
26	3	3	2	1	2	1	3	1	3	2	3	2	1	65.7
27	3	3	2	1	3	2	1	2	1	3	1	3	2	58.6
*k*_1_	57.06	48.66	55.59	57.26	55.84	56.89	56.73	56.03	51.31	56.79	56.92	59.00	56.94	
*k*_2_	57.42	58.38	58.00	57.06	58.98	56.31	57.61	56.41	60.41	55.84	57.68	56.38	58.44	
*k*_3_	57.08	64.52	57.97	57.24	56.73	58.36	57.21	59.11	59.83	58.92	56.96	56.18	56.17	
R	0.37	15.87	2.41	0.20	3.13	2.04	0.88	3.08	9.10	3.08	0.76	2.82	2.28	
Order	B > D > C > A × D > C × D > A													

#### Variance analysis

In order to explore the importance of the influence of the four structural parameters of the straw deflector on scattering performance, combined with the interaction between the factors, the variance analysis was carried out on the simulation test results. In variance analysis, since *MS*_*A*_ < *MS*_*e*_, factor A belongs to the error term, and the corrected difference source *e* was obtained. The results of variance analysis of orthogonal test are shown in Table 4.

**Table 4:** Variance analysis.

Source	SS	df	MS	F	Significance
B	0.114697	2	0.057348	79.90	**
C	0.004614	2	0.002307	3.21	*
D	0.046891	2	0.023446	32.67	**
A × D	0.009398	4	0.002325	3.27	*
$$\left. {\begin{array}{*{20}c} A \\ {C \times D} \\ e \\ \end{array} } \right\}e^{\Delta }$$	$$\left. {\begin{array}{*{20}c} {0.00007} \\ {0.005977} \\ {0.007177} \\ \end{array} } \right\}0.013224$$	$$\left. {\begin{array}{*{20}c} 2 \\ 4 \\ {10} \\ \end{array} } \right\}16$$	0.002247		
SUM	0.188824	26			

*F* checks the critical value in the table (critical value of *F*-test): *F*_0.1_(2,10) = 2.92; *F*_0.05_(2,10) = 4.10; *F*_0.1_(4,10) = 2.61; *F*_0.05_(4,10) = 3.48.

B and D have remarkable significant influence on the test results; Factors C and A × D have significant influence on the test results, but interaction A × D has less influence on the test results than B, D and C. Factors A and C × D had no significant effect on the test results. Therefore, the interaction can be ignored when determining the optimal level of the four factors. The optimal scheme was A_2_B_3_C_2_D_2_, that is, the included angle between the cover straw deflector and the vertical direction was 45°, the height difference between the tail of the cover straw deflector and the outlet was 200 mm, the inclination angle of the inner deflector was 0°, and the inclination angle of the outer deflector was 35°.

#### Visual scattering process analysis

**Figure 10:**
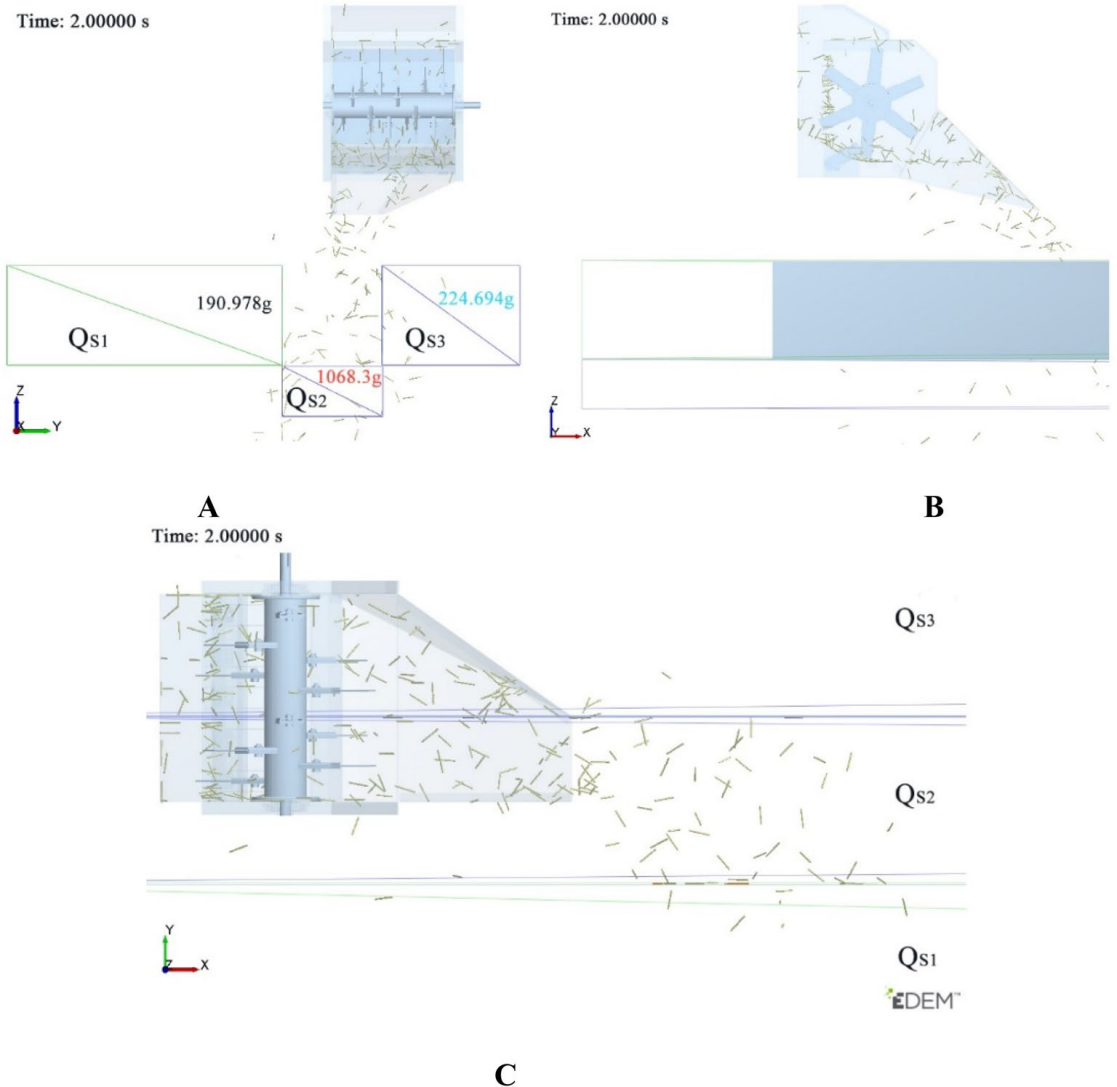
Scattering state of straw at 2 s in the simulation. (**A**) X direction view, (**B**) Y direction view, (**C**) Z direction view.

**Figure 11:**
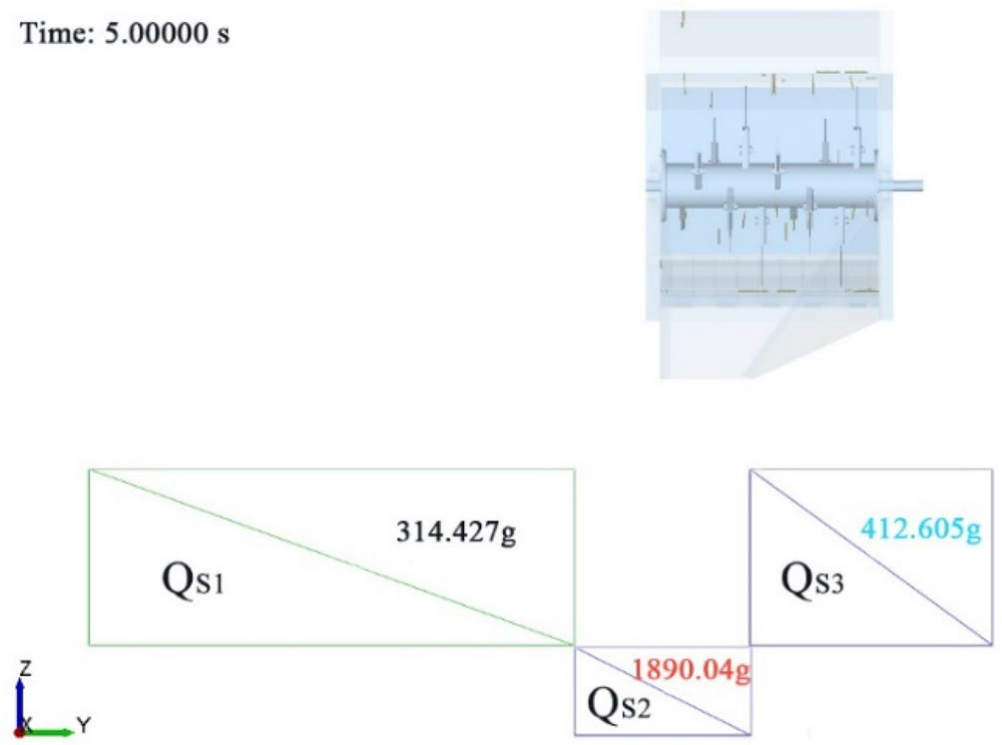
Scattering state of straw at 5 s in the simulation.

The straw scattering process under scheme A_2_B_3_C_2_D_2_ was analyzed. Figure 10 shows the scattering state of smashed straw at the simulation time of 2.0 s, which was relatively stable. In X direction and Z direction view, only a small part of the straw fell into area Q_S1_ and Q_S3_, and the straw falling into these two areas were very close to the simulation baffle II and baffle III, indicating that the displacement (Y direction) of the straw under the guidance of the inner and outer deflector was small, and the scattering range was concentrated. It can be seen from Y direction and Z direction view that a small amount of straw fell near the outlet due to the small initial velocity or falling during the rotation of the knife roller. However, the triangular blank area formed by the outer deflector and the X axis direction was not covered by the bottom plate, so the amount of straw in the Q_S3_ area was greater than that in the Q_S1_ area. In the Y direction view, the scattering distance (displacement in the X direction) of the smashed straw was short. After the smashed straw being discharged from the outlet, they were blocked by the cover deflector and changed the velocity direction. Then, the smashed straw fell to the ground within the angle between the cover straw deflector and the vertical direction. Figure 11 shows the X direction view after the simulation (5.0 s). It can be seen the amount of straw in Q_S1_, Q_S2_ and Q_S3_ was 314.427 g, 1890.04 g and 412.605 g, respectively, and the proportion of straw falling into Q_S2_ was 72.2%. The total amount of straw in the three areas was 2617.072 g, and the total amount of straw generated by the simulation was 3000 g. This was because there were still straw residues in the straw scattering room after the simulation, which was consistent with the actual working condition of the smashing and scattering device.

### Bench verification test results and analysis

**Figure 12:**
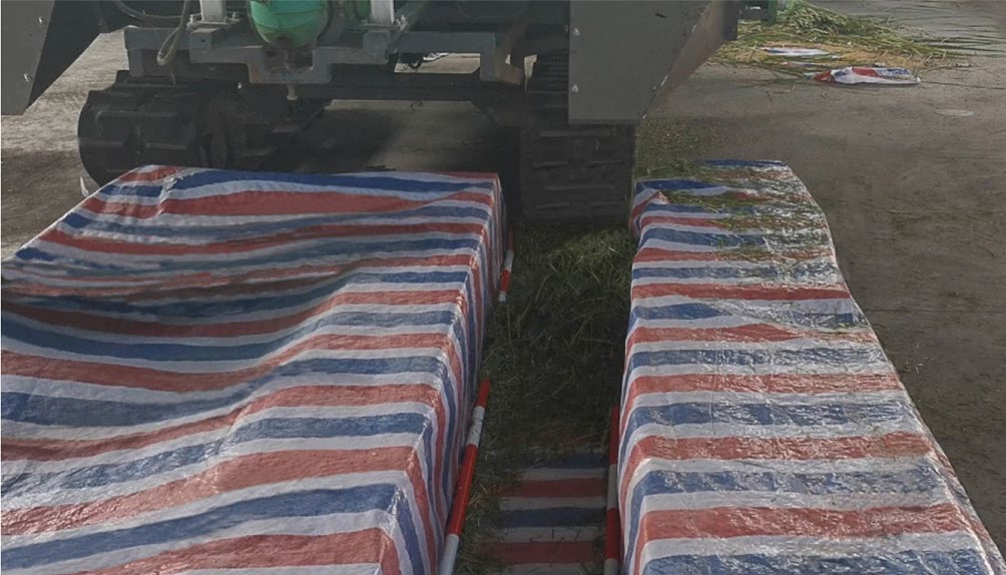
Distribution of straw scattering.

Figure 12 shows the distribution of straw scattered by the smashing and scattering device. As can be seen from the figure, only a small part of the straw was scattered to the right simulated stubble area Q_S3_, and there was no straw in the left stubble area Q_S1_. Most of the straw was scattered to the rolling area Q_S2_. The straw in each area was collected and weighed to obtain the test data as shown in Table 5.

**Table 5:** Quality distribution of crushed straw in different areas.

Serial number	M_*QS*1_/g	M_*QS*2_/g	M_*QS*3_/g	*η*_*QS*_/%
1	0	2655	149	94.7
2	0	2710	186	93.6
3	0	2603	205	92.7
4	0	2584	139	94.9
5	0	2677	228	92.2
Mean value	0	2645.8	181.4	93.6

According to the results of the bench test, the qualified rate of the straw scattering reached 93.6%, while the optimal qualified rate of the simulation test was 72.2%, and the deviation was 21.4%. The main reasons for the deviation are as follows:

1. In the simulation test, the material properties of the established straw particles were fixed, the discrete degree of straw particles was not normal distribution in a complete sense, and there were differences in the material properties among individual straw of ratoon rice in the field harvest progress.
2. In the simulation, only the straw model was established. However, the smashing and scattering device dealt not only the straw, but also with leaves, ear heads and branches, etc. during field harvest. Although these parts did not account for a large amount, they would indeed affect the collision between the smashed particles, and then affect scattering performance.
3. In fact, the straw of ratoon rice is flexible. After being broken, the flexible straw was intertwined with each other and polymerized, and there would be many clumps of smashed straw, whose properties changed greatly, and the force exerted on them was very different from that of a single one.
4. It was observed that the straw scattered into the right simulated stubble area Q_S3_ were all straw fragments that were thoroughly smashed. These fragments were not intertwined before, and were scattered into the area under the action of wind generated by the rotation of the knifer roller and the sweep gas from the harvester engine exhaust pipe. And this straw can fall from the rice stubble to the ground in the actual field harvest, which will cover the rice stubble.

### Field test results and analysis

**Figure 13:**
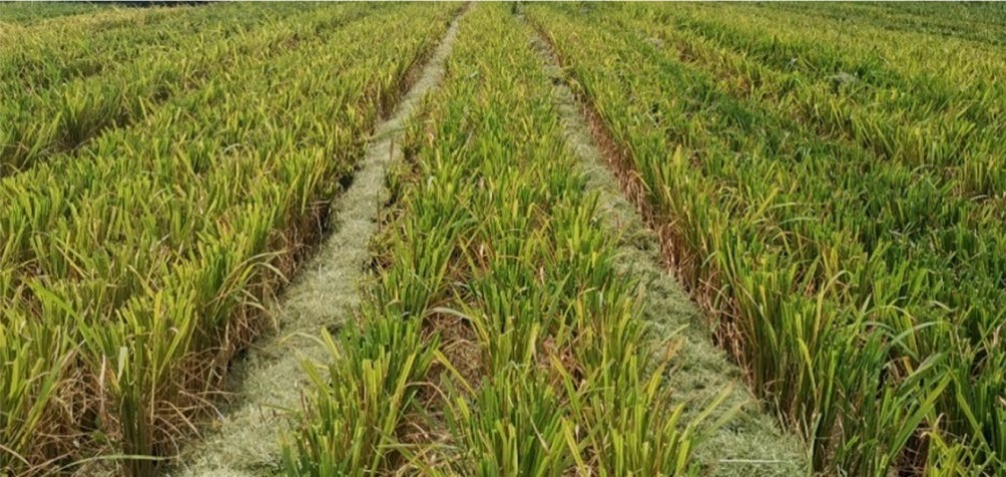
Scattering performance in the field.

Figure 13 shows the straw scattering performance after the harvester operation. According to the test method, the scattering qualified rate was statistically obtained in Table 6. It can be seen from the table that the qualified rate of scattering was as high as 95.2%, and most of the smashed straw could be successfully scattered into the rolling area. Consistent with the results of the bench test, most of the straw collected in the stubble area were fragments, and many of them were not on the surface of the stubble, but on the ground, without covering the stubble.

**Table 6:** Measurement of scattering qualified rate.

Serial number	*M*_*SP*1_/g	*M*_*SP2*_/g	*η*_*PS*_/%
1	5102.7	174.1	96.7
2	5603.0	108.5	98.1
3	5568.2	598.1	90.3
4	6013.1	343.2	94.6
5	5517.3	200.1	96.5
Mean value	5560.9	284.8	95.2

## Conclusions

1. Based on the agrological requirement that the straw of ratoon rice should be smashed and scattered without covering the stubble in the first season harvest, a straw smashing and scattering device matching with the straw scattering requirements of the double-channel feeding ratoon rice harvester was designed in this paper, which could smash the straw from the threshing cylinder and scatter it into the required rolling area.
2. According to the kinematic theory, the force on the straw at the moment when the straw collides with the straw deflector was analyzed. It was found that the angle and size of the straw deflector had a great impact on the straw scattering performance. According to the spatial position relationship between the straw scattering device and the crawler of the double-channel feeding ratoon rice harvester, it was determined that the included angle between the straw deflector and the vertical direction, the height difference between the rear of the straw deflector and the straw outlet, the inclination of the inner plate and the outer plate were the main factors affecting the straw scattering performance.
3. The situation of straw scattering under the action of straw deflector was simulated and analyzed by using EDEM software. It was found that there is a certain interaction between the angle between the straw deflector and the vertical direction and the inclination of the outer plate, the inclination of the inner plate and the inclination of the outer plate, and there was no obvious interaction between other factors. According to the orthogonal test of L_27_ (3^13^), the optimal combination of parameters was obtained as follows: the angle between the cover deflector and the vertical direction was 45°, the height difference between the tail of the cover deflector and the outlet was 200 mm, the inclination angle of the inner deflector was 0°, and the inclination angle of the outer deflector was 35°. The qualified rate of scattering simulation was 72.2%.
4. According to the optimal combination of structural parameters obtained from the simulated orthogonal test, the qualified rate of the straw scattering reached 93.6% in the bench test, and the qualified rate of the comprehensive straw scattering reached 95.2% in the field test when it was fit with the double-channel feeding ratoon rice harvester, which could meet the harvest demand of the first season of ratoon rice. The smashed straw could basically be discharged to the two crawler rolling areas of the harvester to meet the harvest requirement of ratoon rice in the first season and facilitate the ear germination of ratoon rice in the second season.
5. It can be seen from the results of the three groups of tests that although there was a big difference between the results of the simulation test and the bench test, the optimal parameter combination of the straw deflector selected by the simulation test did have a remarkable performance. Therefore, it is feasible to use EDEM software to simulate the process of straw scattering to study the influence of structural parameters of straw deflector on the performance of straw scattering.

## Data Availability

The datasets used and/or analysed during the current study available from the corresponding author on reasonable request.

## References

[1] Mo W (2020). Effects of planting patterns and varieties on quality and yield of ratooning and late season rice.

[2] Lin X (2019). Model optimization and application potential evaluation of process-based ratoon rice growth simulation model.

[3] Wang F, Peng S (2018). Research progress in rice green and high-yield management practices. Chin. Bull. Life Sci..

[4] Dong H, Chen C, Wang W, Peng S, Hang J, Cui K, Nie L (2017). The growth and yield of a wet-seeded rice-ratoon rice system in central China. Field Crop Res..

[5] Mao S (2021). Studies Effects of different irrigation and nitrogen application treatments on axillary bud growth and grain yield formation of ratoon rice.

[6] Qian Q, Guo L, Smith SM, Li J (2016). Breeding high-yield superior quality hybrid super rice by rational design. Natl. Sci. Rev..

[7] Deng Q (2019). Effects of Cultivation Modes on Soil Fertility, Greenhouse Gases Emission and Yield in Ratoon Rice Fields.

[8] Dong C, Xu N, Ding C, Gu H, Zhang W, Sun L (2020). Developing ratoon rice as forage in subtropical and temperate areas. Field Crop Res..

[9] Wang H, Zhang Q, Zhang W, Li S, Huang J, Zhu A, Liu L (2020). Advances in the effects of the ability of axillary bud germination on grain yield in ratoon rice. Chin J Rice Sci..

[10] Duan X, Zhang W, Yao X, Li J, Tang Y, She X, Xiao R (2019). High-yielding and high-efficiency cultivation techniques for ratooning rice of mid-season hybrid rice under mechanical harvest conditions. HybridRice..

[11] Jiang Z, Zheng J, Shen R, Xie Z, Yu D, Li Y, Huang Y, Wang H (2018). Germination of nodal roots of ratooning tillers of ratoon rice cultivar Jiafuzhan harvested by low stubble machine-cut. J. Xiamen Univ. (Nat. Sci.)..

[12] He A, Wang W, Jiang G, Sun H, Jiang M, Man J, Cui K, Huang J, Peng S, Nie L (2019). Source-sink regulation and its effects on the regeneration ability of ratoon rice. Field Crop Res..

[13] Wang X (2018). Effects of different nitrogen managements on bud growth and nitrogen utilization characteristics in ratoon rice. MS thesis.

[14] Ma X (2015). Studies on variety screening ratoon rice and the effect of main crop harvesting model on ratoon crop yield.

[15] Zhang Z, He J, Li H, Wang Q, Ju J, Yan X (2017). Design and experiment on straw chopper cum spreader with adjustable spreading device. Trans. Chin. Soc. Agric. Mach..

[16] Sun N, Wang X, Li H, He J, Wang Q, Wang J, Liu Z, Wang Y (2019). Design and experiment of differential sawing rice straw chopper for turning to field. Trans. Chin. Soc. Agric. Eng. (Trans. CSAE).

[17] Qin K, Cao C, Liao Y, Wang C, Fang L, Ge J (2020). Design and optimization of crushing and throwing device for straw returning to field and fertilizing hill-seeding machine. Trans. Chin. Soc. Agric. Eng. (Trans. CSAE).

[18] Thakur SS, Garg IK (2007). Paddy straw management by chopping for sowing wheat in combine harvested field. J. Res..

[19] Schillinger WF, Smith TA, Schafer HL (2008). Chaff and straw spreader for a plot combine. Agron. J..

[20] Lisowski A, Świątek K, Klonowski J, Sypuła M, Chlebowski J, Nowakowski T, Kostyra K, Strużyk A (2012). Movement of chopped material in the discharge spout of forage harvester with a flywheel chopping unit: Measurements using maize and numerical simulation. Biosys. Eng..

[21] Fu J, Xie G, Ji C, Wang W, Zhou Y, Zhang G, Zha X, Anwer AM (2021). Study on the distribution pattern of threshed mixture by drum-shape bar-tooth longitudinal axial flow threshing and separating device. Agriculture.

[22] Fu J, Zhang G, Xie G, Wang Y, Gao Y, Zhou Y (2020). Development of double-channel feeding harvester for ratoon rice. Trans. Chin. Soc. Agric. Eng. (Trans. CSAE)..

[23] Zheng Z, He J, Wang Q, Li H, Li W, Chen W (2017). Design and experiment on straw pickup-chopping and ditch-burying integrated machine. Trans. Chin. Soc. Agric. Mach..

[24] He Y, Wu M, Xiang W, Yan B, Wang J, Bao P (2017). Application progress of discrete element method in agricultural engineering. Chin. Agric. Sci. Bull..

[25] Wang G, He W, Wang J (2010). Discrete Element method and its practice on EDEM.

[26] Zhang C (2019). Design and Experiment of Key Components of Six-Head Spiral Straw Returning Cultivator.

[27] Lu K (2017). Design and Performance Experiment of a Small Horizontal-Axial Threshing and Separating Device for Ratoon Rice.

[28] Li Y, Hu C (2017). Experimental Design and Data Processing.

[29] Zhu Q, Wu G, Chen L, Zhao C, Meng Z (2018). Influences of structure parameters of straight flute wheel on fertilizing performance of fertilizer apparatus. Trans. Chin. Soc. Agric. Eng. (Trans. CSAE)..

[30] GB/T 24675.6–2009, Conservation tillage equipment—Smashed straw machine, https://max.book118.com/html/2022/0208/8102016042004056.shtm.

